# Toolbox to Reduce Lumpectomy Reoperations and Improve Cosmetic Outcome in Breast Cancer Patients: The American Society of Breast Surgeons Consensus Conference

**DOI:** 10.1245/s10434-015-4759-x

**Published:** 2015-07-28

**Authors:** Jeffrey Landercasper, Deanna Attai, Dunya Atisha, Peter Beitsch, Linda Bosserman, Judy Boughey, Jodi Carter, Stephen Edge, Sheldon Feldman, Joshua Froman, Caprice Greenberg, Cary Kaufman, Monica Morrow, Barbara Pockaj, Melvin Silverstein, Lawrence Solin, Alicia Staley, Frank Vicini, Lee Wilke, Wei Yang, Hiram Cody

**Affiliations:** Gundersen Health System Norma J. Vinger Center for Breast Care, La Crosse, WI USA; David Geffen School of Medicine, University of California Los Angeles, Burbank, CA USA; Morsani College of Medicine, University of South Florida, Tampa, FL USA; Dallas Surgical Group, Dallas, TX USA; City of Hope Medical Group, Rancho Cucamonga, CA USA; Mayo Clinic, Rochester, MN USA; Baptist Cancer Center, Baptist Memorial Health Care System, Memphis, TN USA; New York Presbyterian Hospital, New York, NY USA; Columbia University, New York, NY USA; Mayo Clinic, Owatonna, MN USA; School of Public Health and Medicine, University of Wisconsin Madison, Madison, WI USA; Bellingham Breast Center, Bellingham, WA USA; Memorial Sloan Kettering Cancer Center, New York, NY USA; Weill Cornell Medical College, New York, NY USA; Mayo Clinic, Scottsdale, AZ USA; Hoag Memorial Hospital, Newport, CA USA; Keck School of Medicine, University of Southern California, Los Angeles, CA USA; Albert Einstein Healthcare Network, Philadelphia, PA USA; Akari Healthcare, Boston, MA USA; 21st Century Oncology, St. Joseph Mercy Oakland, Pontiac, MI USA; MD Anderson Cancer Center, University of Texas, Houston, TX USA

## Abstract

**Background:**

Multiple recent reports have documented significant variability of reoperation rates after initial lumpectomy for breast cancer. To address this issue, a multidisciplinary consensus conference was convened during the American Society of Breast Surgeons 2015 annual meeting.

**Methods:**

The conference mission statement was to “reduce the national reoperation rate in patients undergoing breast conserving surgery for cancer, without increasing mastectomy rates or adversely affecting cosmetic outcome, thereby improving value of care.” The goal was to develop a toolbox of recommendations to reduce the variability of reoperation rates and improve cosmetic outcomes. Conference participants included providers from multiple disciplines involved with breast cancer care, as well as a patient representative. Updated systematic reviews of the literature and invited presentations were sent to participants in advance. After topic presentations, voting occurred for choice of tools, level of evidence, and strength of recommendation.

**Results:**

The following tools were recommended with varied levels of evidence and strength of recommendation: compliance with the SSO-ASTRO Margin Guideline; needle biopsy for diagnosis before surgical excision of breast cancer; full-field digital diagnostic mammography with ultrasound as needed; use of oncoplastic techniques; image-guided lesion localization; specimen imaging for nonpalpable cancers; use of specialized techniques for intraoperative management, including excisional cavity shave biopsies and intraoperative pathology assessment; formal pre- and postoperative planning strategies; and patient-reported outcome measurement.

**Conclusions:**

A practical approach to performance improvement was used by the American Society of Breast Surgeons to create a toolbox of options to reduce lumpectomy reoperations and improve cosmetic outcomes.

A gap in quality of healthcare exists whenever variability of care coexists with evidence that high performance is achievable.^1^,^2^ Multiple, recent reports have documented significant variability of care for oncologic reoperation after initial lumpectomy for breast cancer.^3^–^6^ Rates of reoperation vary from less than 10 % to more than 50 %. This variability is not accounted for by patient or disease characteristics. Therefore, the American Society of Breast Surgeons (ASBrS) convened a multidisciplinary consensus conference entitled a “Collaborative Attempt to Lower Lumpectomy Reoperation rates” (CALLER).

The CALLER conference mission statement was defined as: “Reduce the national reoperation rate in patients undergoing breast-conserving surgery for cancer, without increasing mastectomy rates or adversely affecting cosmetic outcome, thereby improving value of care.”

The purpose of the consensus conference was to develop a practical toolbox of recommendations to help providers reduce lumpectomy reoperations to the best achievable level based on available evidence and expert opinion. The target goal is not zero, and to attempt this would be expected to impact cosmetic outcome and lower the breast-conserving therapy rate. The group identified and considered concurrent efforts to reduce reoperation variability, including the meta-analysis that resulted in the SSO-ASTRO margin statement and an updated systematic review of the literature performed by the American College of Surgeons for their new “Operative Standards for Cancer” manual.^7^–^9^

## Methods

Consensus conference participants included experts in breast cancer care from multiple disciplines (surgery, radiology, pathology, plastic surgery, and radiation and medical oncology). A statistician and a patient representative with patient advocacy experience were included. Participants with expertise in quality measurement, patient-reported outcomes, guideline development, and clinical trials were present. There was diversity across breast surgeon practice type, including community and academic surgeons.

Toolbox development followed to the extent possible the standards of the Institute of Medicine for guideline development.^10^ Multiple recent systematic literature reviews were referenced by participants.^7^,^9^,^11^–^21^ Before the conference, all participants were provided with key topics, references, speaker presentations, and potential “tools” for the toolbox. After topic presentation, an interactive discussion occurred followed by voting. Conference participants and the ASBrS Board of Directors approved toolbox recommendations.

## Results

The proposed conference tools, references, level of evidence, consensus, and strength of recommendation are described in Tables 1 and 2. Recognizing the impact of reoperations on patient care, cost, and outcomes, the conference participants had uniform agreement to set a 5-year target goal for a national average reoperation rate in the year 2020. However, there was lack of uniformity for the actual target number. Two-thirds (10/15) of participants recommended a goal of less than 20 %.

**Table 1:** CALLER Toolbox to reduce reoperation and improve cosmetic outcomes

Tool	% CALLER participants recommending	Level of evidence/consensus	Strength of recommendation	References
SSO-ASTRO^a^ guideline	94 %	High 2A nonuniform	Strong-moderate	7,8,14
Minimally invasive breast biopsy	94 %	High 1 nonuniform	Strong	12,15,49,50
Complete diagnostic mammography and US as needed	94 %	Lower 2B nonuniform	Strong-moderate	11,16,51–54
Oncoplastic lumpectomy	100 %	Lower 2A uniform	Strong-moderate	17,43–48,55,56
Lesion localization	94 %	Lower 2A nonuniform	Strong	9,18–20,49,50,53,54,57–86
Specimen orientation	95 %	Lower 2A nonuniform	Strong	49,50,87,88
Cavity shaves	75 %	Lower 2A nonuniform	Strong-moderate	25,89–97
Specimen imaging and surgeon review	100 %	Lower 2A uniform	Strong	50,98–106
Intraoperative pathology	89 %	Lower 2A–2B nonuniform	Strong-moderate	13,21,27,107–124
Preoperative multidisciplinary planning	100 %	Lower 2A uniform	Strong-moderate	49,50,125,126
Patient-reported outcome measurement	57 %	Lower 2B nonuniform	Moderate-weak	127–135

^a^SSO-ASTRO guideline only applicable for invasive cancer

**Table 2:** Level of evidence/consensus and strength of recommendation categories

Strength of recommendation	Level of evidence/consensus
1. Strong 2. Strong-moderate 3. Moderate 4. Moderate to weak 5. Weak 6. Insufficient evidence	1. (1) High-level evidence; uniform CALLER consensus that intervention is appropriate 2. (2A) Lower-level evidence; uniform CALLER consensus that intervention is appropriate 3. (2B) Lower-level evidence; CALLER majority consensus that intervention is appropriate 4. (3) Based on any level evidence; major CALLER disagreement that intervention is appropriate

Level of evidence and consensus scale is adapted from NCCN guidelines

### Tool 1: Preoperative Diagnostic Imaging Should Include Full-Field Digital Mammography and Supplementary Imaging to Include Ultrasound as Needed

All participants agreed that high-quality, meticulous, preoperative, diagnostic mammography was necessary preoperatively. “Selective” use of ipsilateral ultrasound (US) was recommended. US may be of less benefit when screening mammography identifies calcifications without mass. Despite near routine actual use of US by conference participants, they concluded that the level of evidence did not support a recommendation for “routine” US. Breast tomography was discussed and judged to have future applications but was not yet included in the toolbox due to insufficient evidence. Routine use of MRI was not recommended based on meta-analyses that show its use does not affect the rate of reexcision or local recurrence. Selective use of MRI is described in position statements from other groups.^22^–^24^

### Tool 2: Minimally Invasive Breast Biopsy (MIBB) for Breast Cancer Diagnosis

Some studies demonstrate lower reoperation rates when a diagnosis of malignancy is known before surgical excision. MIBB provides opportunity for preoperative treatment planning to include genetic risk assessment, medical oncology, and plastic surgery consultation and axillary evaluation.

### Tool 3: Multidisciplinary Discussions to Include Radiology, Pathology, Surgery, and Radiation and Medical Oncology

Optimizing reoperation rates requires preoperative collaboration between radiologists, surgeons, and pathologists. In patients considered for neoadjuvant therapy, medical oncology consultation also is necessary. Preoperative knowledge of number of lesions, geometry, distance to skin and chest wall, and possible extension towards the nipple may all facilitate negative margins. Information technology that enhances communication and provides intraoperative archived images can aid lesion review and communication. Postoperative discussion with all specialties aids decision making regarding reoperation.

### Tool 4: For Nonpalpable Breast Lesions, the Use of Radioactive Seeds, Intraoperative US, or Wire Localization to Direct Lesion Excision is Recommended

A localization method should be used for resection of all nonpalpable cancers. Although some studies have indicated superiority of one technique compared with another, the conference concluded that evidence to recommend a single technique was not definitive. Surgeon use of US also can be used to aid targeting and decide volume of resection in both palpable and nonpalpable lesions. Placement of multiple localizing wires or seeds (bracketing) may be useful for larger lesions, multifocal tumors, or extensive ductal carcinoma in situ (DCIS).

### Tool 5: Oncoplastic Techniques can Reduce the Need for Reoperation in Anatomically Suitable Patients

Oncoplastic techniques have the potential to decrease positive margins at initial lumpectomy by allowing resection of a larger volume of tissue. They also may improve ipsilateral breast appearance and contralateral breast symmetry. There was uniform agreement for their potential benefit. The conference recommends applying these techniques only in a selective group of patients. Small primary cancers can be excised with acceptable cosmetic results without oncoplastic techniques. For all procedures, marker clips or other marking modality should be considered for application to cavity side walls to aid radiation planning.

### Tool 6: Specimen Orientation of 3 or More Margins

When the breast cancer is excised, markers or ink should be placed on the specimen for orientation to ensure which margin edge(s) is/are positive to guide focused reexcision of the correct tissue, if necessary. There are limited data linking orientation directly to reoperation rates, but the conference concluded the benefit/burden ratio of orientation was high. All excisions should be oriented. Orientation is associated with better cosmetic outcomes by avoiding “entire cavity” reexcision in patients with nonoriented positive margins. The consensus was that orientation of at least three sides was superior to two sides. Some participants favored intraoperative six-sided inking as best practice, but there was no consensus on orientation methodology beyond labeling at least three margins.

### Tool 7: Specimen Radiograph with Surgeon Intraoperative Review

The primary role of specimen imaging is to document removal of the targeted nonpalpable lesion before the patient leaves the operating room. Lower-level evidence supports specimen radiography as a method to assess distance of lesion to margin and therefore direct and potentially reduce reoperation. Specimens should not undergo compression during imaging, because it may cause specimen fracture that allows ink to enter the crevasse and a false-positive margin. Some participants supplement specimen radiography with US. Surgeons should review the specimen imaging before the operation has been completed, ideally with surgeon-radiology communication. Real-time review may avoid a complete “miss” of the lesion or direct the surgeon to perform an additional cavity shave for a “close” margin. Specimen imaging may not be universally available. If not, the conference strongly encourages systems to develop necessary resources for specimen imaging with immediate image review. Two views at orthogonal angles may identify close or positive margins not seen on a single view. Intraoperative imaging with other modalities to include tomograms, MRI, CT, and other imaging are being investigated.

### Tool 8: Consider Cavity Shave Margins in Patients with T2 or Greater Tumor Size or TI with Extensive Intraductal Carcinoma (EIC)

There are moderate levels of evidence that cavity side wall excisions correlate with lower reoperation rate. Shave size should provide adequate sampling of the residual wall. “Tiny shaves” representing only a small portion of a “wall” were discouraged. If shaves are performed, the “final” edge should be marked; i.e., nonoriented shave with even a small amount of tumor on the surface would constitute a final ink positive margin status requiring reexcision. Some surgeons routinely perform shaves of all cavity side walls regardless of tumor type or size. Others perform selective shaves directed by palpation, imaging, or pathologic specimen examination. There has been one recently published, randomized, controlled trial of cavity shave versus no-shave margins, which demonstrated a statistically significant decrease in the reoperation rate for patients undergoing breast conservation surgery.^25^

### Tool 9: Intraoperative Pathology Assessment of Lumpectomy Margins may Help Decrease Reexcision When Feasible

A systematic literature review demonstrates that intraoperative margin assessment with frozen histologic section or imprint cytology are associated with lower reoperation rates by allowing intraoperative reexcision of positive margins.^13^ There is lower-level evidence to support only gross specimen examination. Resources and expertise may limit the feasibility of routine intraoperative pathology assessment. Several institutions report low reoperation rates without intraoperative margin assessment.

### Tool 10: Compliance with the SSO-ASTRO Margin Guideline to Not Routinely Reoperate for Close Margins with no Tumor on Ink in Patients with Invasive Cancer

Compliance with this guideline has the potential to reduce reoperations by 40 %.^6^ The remaining tools are targeted towards reducing ink positive margins at the initial lumpectomy. By meta-analysis, recurrence risk doubles when ink positive margins are not excised. Recurrence is not improved by reoperation if the margin is negative. If ink positive margins occur, the need for reoperation should be evaluated by the treating team in collaboration with the patient (“shared decision making”), providing patients with recurrence risks in absolute percentages for the choices of reoperation or not. As a consequence, some patients may choose not to have reoperation. The margin guideline is applicable to subsets of patients with “bad tumor biology” (triple negative, Her 2 positive, high grade), young age, lobular cancer, EIC, or not receiving systemic treatment. There is no proven benefit for reoperation in these patients if they have ink negative margins. Some patients with negative margins may still be considered for reoperation, if clinical and/or imaging findings suggest residual persistent adjacent disease. The margin meta-analysis did not include patients with neoadjuvant therapy or pure DCIS. Given the lack of consensus regarding acceptable margin width for DCIS, decisions regarding reoperation in these patients optimally involves multidisciplinary input and shared decision making with the patient. Until new evidence is available for DCIS, the conference supports NCCN guidelines for reoperation if the margin is ink positive or <1 mm.^26^

### Tool 11: Routine Breast-Specific Patient Reported Outcome (PRO) Measurement may Help to Assess Cosmetic Outcomes When Feasible

There is limited reporting in the literature of cosmetic and functional outcomes from the patient perspective. Validated PRO tools, such as BREAST-Q^©^, should be more widely adopted and may aid improvement. New tools need to be developed that decrease the burdens for both providers and patients for reporting.

## Discussion

The goal of the consensus conference was to provide practitioners with a variety of tools that can be adapted to help lower rates of reoperation following lumpectomy. While these recommendations are not meant to serve as guidelines or standard of care, conference leaders complied with most principles for guideline development as defined by the IOM.^10^ Updated systematic reviews were referenced and the group included multiple disciplines and stakeholders.^7^,^9^,^11^–^21^ The group did not provide a period for public comment, request for other society endorsement, or commission new systematic literature reviews. For expediency, recommendations were provided that could be implemented into clinical practice quickly. “Standard of care” is a legal term, and our toolbox does not establish a new legal “standard of care.” It also is important to recognize that performing reoperation does not mean poor quality care. Particularly, omission of reoperation for positive margins is not recommended. Reoperation of a positive margin is good quality care and results in lower risk of cancer recurrence. All tools in the toolbox earned endorsement by a majority vote. It does not follow that all tools are recommended for every patient.

At least three factors should be considered for selection. The first is resource availability. For example, one tool is the use of intraoperative frozen section (FS) for margin assessment, a tool associated with very low rates of reoperation.^27^ This service may not be available in all settings, and there should be no inference of “poor quality” for lack of access to it. In contrast, multidisciplinary preoperative planning—in person or virtual—can be implemented widely.

The second consideration for tool selection is baseline reoperation rate. The average reoperation rate in four national databases ranges from 20 to 24 %.^3^–^6^ For surgeons and institutions with average or higher rates, a trial of previously unused or underutilized tools should be considered, followed by tracking of outcomes. For those with rates already in the best tiers of performance, there can be attempts to improve even further by testing different or additional tools, but performance tracking will still be necessary.

The last consideration for number of tools is “redundancy.” For example, if circumferential lumpectomy FS is used and negative, then the benefit of additional shaving of cavity side walls is low. If complete cavity side wall shavings are performed, then the benefit of lumpectomy margin FS is low too. Some participants recommended using more tools when operating on patients with known factors associated with positive margins, such as larger size, invasive lobular type, low-grade noncalcified DCIS, and EIC status. All tools in the toolbox can be applied for patients with DCIS and invasive cancer except the SSO-ASTRO margin statement, which was specific for invasive cancer and did not include patients with pure DCIS.

Intraoperative devices to assess margin status were discussed as potential tools to decrease reoperation. A recent, randomized trial concluded that the MarginProbe™ device was associated with fewer reoperations.^28^ The conference majority vote was to omit these devices from the toolbox until further investigation.^28^–^36^

Measurement of both individual surgeon and institutional outcomes are essential prerequisites during attempts to reduce reoperation after initial lumpectomy. Measurement assesses the impact of these initiatives. If resources are available, a comprehensive audit that tracks intended and unintended outcomes is recommended (Table 3). If resources are limited, then minimal tracking would include reoperation, positive margin, and breast-conserving therapy (BCT) rates. Reoperation rates and BCT rates can be reported in the ASBrS Mastery database, the National Consortium of Breast Centers Quality Measurement Program, and “in-house” registries.^37^,^38^ All breast cancer quality-measurement programs were recently summarized.^39^

**Table 3:** Performance tracking options during initiatives to reduce reoperation and improve cosmetic outcome

1. Core needle biopsy rate for cancer diagnosis*
2. Specimen imaging rate*
3. Specimen orientation rate*
4. Rate of ink positive margins at initial lumpectomy
5. Compliance rate with SSO-ASTRO margin statement
6. Reoperation rate after initial lumpectomy for breast cancer*
7. Breast conserving therapy rate
8. Cost/charges per episode of care
9. Patient reported outcomes to include cosmetic outcome after lumpectomy*
10. Ipsilateral breast tumor recurrence rate

* ASBrS endorsed Quality Measure audited in Mastery Program^37^

Increased mastectomy rates and poor cosmetic outcomes are potential unintended adverse outcomes of efforts to lower reoperation rates and therefore should be monitored.^40^–^42^ These risks were recognized but were felt to be balanced by the potential to improve overall patient care by following conference recommendations. There is evidence that both reoperation rate and cosmetic outcome can improve by adoption of oncoplastic techniques.^43^–^48^

The conference process and work product is not without limitations. We did not follow strict guideline development standards and did not use a formal Delphi process in arriving at consensus. Furthermore, most of the tools are not based on high-level evidence. The strength of the conference is its recognition that unacceptable variability occurs in the care of patients undergoing lumpectomy. As a consequence, multiple stakeholders accepted ownership and then developed recommendations to improve care, cost, and outcomes by using “best available” evidence and expert opinion.

## Conclusions

Recognition of the gap between actual and achievable care led to development of a toolbox of recommendations to reduce the proven variability of reoperation and the suspected variability of cosmetic outcome after initial lumpectomy for breast cancer. A list of other ASBrS initiatives to reduce reoperation and improve cosmesis is described in Table 4. Tracking of outcomes is recommended for all initiatives. Next steps include: (1) dissemination and implementation strategies; (2) comparative effectiveness research to determine which tools or collection of tools are most strongly associated with reoperation rates, cosmetic outcome, and value; and (3) collaboration with industry, payer, and government stakeholders to provide better support for performance reporting that is funded, incentivized, and less burdensome for providers.

**Table 4:** American Society of Breast Surgeon efforts to reduce variability of reoperation rates after initial lumpectomy for cancer

1. Orlando Consensus Conference April 30, 2015
2. Auditing and peer performance comparison of re-excision rates and reasons for re-operation available in the ASBrS Mastery Program^6^,^37^
3. Development of formal specifications for a reexcision lumpectomy rate quality measure in 2014
4. Development of a patient reported cosmetic outcome measure in the Mastery patient survey
5. Development of a guideline for the technique of “breast-conserving surgery” available on the ASBrS website
6. Education emphasizing compliance with the SSO-ASTRO margin statement during the 2014 and 2015 annual meetings
7. Quality and Research committees of the ASBrS to begin a prospective, observational study of members to search for associations between reoperation rates and the CALLER conference tools in 2015. This effort is intended to aid the design of subsequent comparative effectiveness research
